# Medical Schools and Students Abroad

**Published:** 1861-05

**Authors:** 


MEDICAL SCHOOLS AND STUDENTS ABROAD.—During the past winter, the number of students in Paris was 1156; in London, 1237; and in Dublin, 806; showing a large increase over the session of 1859-60. In Dublin, the number attending the different schools was as follows:—Ledwich School, 228; College of Surgeons' School, 220; Cecilia Street School, 101; Trinity College School, 100; Richmond Hospital School, 97; Steevens' Hospital School, 60.

